# Effect of Thermomechanical Loading at Low Temperatures on Damage Development in Glass Fiber Epoxy Laminates

**DOI:** 10.3390/ma17010016

**Published:** 2023-12-20

**Authors:** Anna Krzak, Zainab Al-Maqdasi, Agnieszka J. Nowak, Roberts Joffe

**Affiliations:** 1 Scientific and Didactic Laboratory of Nanotechnology and Material Technologies, Silesian University of Technology, 44-100 Gliwice, Poland; agnieszka.j.nowak@polsl.pl; 2 Department of Engineering Sciences and Mathematics, Luleå University of Technology, 97187 Luleå, Sweden; zainab.al-maqdasi@ltu.se (Z.A.-M.); roberts.joffe@ltu.se (R.J.)

**Keywords:** laminates, low temperature, stiffness degradation, epoxy/glass, mechanical test

## Abstract

Due to the high interest in the use of glass/epoxy laminates in aerospace applications, aviation, and as cryogenic tanks, it is crucial to understand the behavior of composites under challenging environmental conditions. Polymer composites are exposed to low temperatures, including cryogenic temperatures, which can lead to the initiation of microdamage. This paper investigates damage initiation/accumulation and its influence on the properties of cross-ply woven glass fiber epoxy composites at low temperatures compared to room temperature conditions. To evaluate the influence of a low-temperature environment on the mechanical performance of glass fiber reinforced epoxy composite (GFRP) laminates, three types of test campaigns were carried out: quasi-static tensile tests and stepwise increasing loading/unloading cyclic tensile tests at room temperature and in a low-temperature environment (−50 °C). We demonstrated that the initial stiffness of the laminates increased at low temperatures. On the other hand, there were no observed changes in the type or mechanism of developed damage in the two test conditions. However, the reduction in stiffness due to the accumulated damage was more significant for the laminates tested at low temperatures (~17% vs. ~11%). Exceptions were noted in a few formulations where the extent of damage at low temperatures was insignificant (<1%) compared to that at room temperature. Since some of the studied laminates exhibited a relatively minor decrease in stiffness (~2–3%), we can also conclude that the formulation of matrix material plays an important role in delaying the initiation and formation of damage.

## 1. Introduction

In recent years, research has focused on analyzing the mechanical behavior of polymer matrix composites at low and cryogenic temperatures. Glass/epoxy composites are promising materials for cryogenic applications and have attracted the attention of many scientists. Laminates with intricate arrangements are commonly employed to achieve high stiffness. However, under operational conditions, laminates are subjected to complex thermomechanical loads that lead to the accumulation of damage. Matrix cracking or transverse cracking is one of the primary types of damage that can occur in multi-axial laminates. This type of damage can result in catastrophic consequences in certain situations, such as leaks in pressure vessels, rendering it entirely unacceptable. Conversely, in structures where only stiffness is a crucial concern, this type of damage may cause some stiffness degradation, which can be tolerated to some extent. To ensure the proper design of structures, it is imperative to understand the failure mechanism and to predict the degree of degradation of the mechanical properties [1]. Among all types of reinforcements, glass and carbon fabrics (CRRPs) are the preferred choice in the aerospace industry due to their excellent properties (high strength, stiffness, and fatigue resistance) [2]. Shokrieh [3], Hohe [4], Zsombor [5], and Chen [6] reviewed the properties of composite materials for cryogenic applications. Their comprehensive overview covers research advancements in polymers for cryogenic applications, as well as the thermal, mechanical, and electrical behavior of these materials. Zsombor Sapi [5] defined the temperature ranges for cryogenic, low temperature, and room temperature conditions. Cryogenic temperature (CT) ranges from −273 °C to −150 °C, low temperature (LT) ranges from −150 °C to −50 °C, and room temperature (RT) is around 23 °C.

Many works devoted to the tensile testing of composite materials, encompassing experimental and analytical studies conducted under cryogenic conditions, can be found in the literature. Torabizadeh [7] conducted experiments on UD glass-fiber-reinforced polymer composites under tensile, compressive, and shear loads at low temperatures. The results showed that low temperatures notably impact the failure mode. Yuanchen Li [8] designed innovative cryogenic in-situ tensile tests with micro X-ray-computed tomography (μCT) to study the microstructure and morphological evolution of woven fabric composites (WFCs). The results indicate that initiating multiple transverse cracks within the weft yarns and delamination damage are the primary microscale characteristics of glass/epoxy WFCs at low temperatures. Perez [9] presented a detailed report on tensile and compressive testing at −165 °C using fixtures and loading devices typically used for testing at room temperature or elevated test temperatures. In another study, Kumarasamy [10] investigated the tensile strength of glass-fiber-reinforced polymer (GFRP) composites at high and low temperatures. The tensile strength and Young’s modulus were measured at room temperature as 275 MPa and 8 GPa, respectively. Under cryogenic conditions, Kumarasamy [10] observed an increase in tensile strength to 315 MPa. On the other hand, Anjaneyulu [11] fabricated composites using epoxy resin with various orientations of glass fibers and presented studies on the effect of symmetrical and non-symmetrical fiber orientations on the tensile and flexural properties of epoxy laminates reinforced with unidirectional E-glass fibers. LeBlanc [12] analyzed the effect of low temperatures on the mechanical properties, cracking, impact strength, and dynamics of carbon/glass and epoxy composites. The E-glass/epoxy-based material that was tested showed minimal dependence on temperature drops regarding its elastic modulus and tensile strength. A team of Bhavya scientists [13] developed an epoxy/glass laminate using various curing agents, which was tested for its tensile, flexural, shear, and impact properties. In analyzing the mechanical, dielectric, and thermal parameters, it was concluded that the composite with diethyl toluene diamine hardener was a better choice than the composite with triethylenetetramine as a hardener for use as an electromagnetically transparent mechanical coating for the antenna system. Takeda [14] used finite element analysis to study G-11 fiberglass/epoxy laminates, considering the presence of cracks and temperature-dependent tensile properties, at extremely low temperatures (4K). The findings indicate that cracked laminates have a lower Young’s modulus compared to non-cracked ones, with surface cracks leading to a notable reduction. Shindo [15] described experimental and analytical studies of the cryogenic fatigue behavior of woven polymer laminates reinforced with glass fibers when subjected to load in Mode I. They found that, at low temperatures, fatigue load caused damage in the form of fiber cracking and matrix cracking, leading to degradation of the material properties. Also, Shindo [16] experimentally and numerically investigated the cryogenic fatigue delamination behavior of woven glass-fiber-reinforced polymer laminates under mode I loading. The results showed that the fatigue delamination growth rate of GFRP woven laminates at low temperatures was significantly lower than at room temperature. Kanno [17] studied the stresses experienced by polymer matrix composite materials under tensile and shear loading using numerical and experimental methods. The tests were carried out at different stacking angles at room temperature. It was confirmed that the interlayer tensile strength decreased, whereas the interlayer shear strength increased with an increasing lay angle. The Tefer research group’s article [18], which is part of an ongoing study on the durability and performance of FRP materials in wind turbine blade construction, discusses experimental and analytical investigations into the mechanical behavior of glass-fiber-reinforced polymer laminates at various temperatures. They confirmed that low temperatures lead to an increase in Young’s modulus.

There are few reports in the scientific literature investigating the effect of low and cryogenic temperatures on the mechanical and thermal properties of various epoxy matrix compositions. Ueki [19] compared the mechanical and thermal properties of several types of epoxy systems that were to be used under cryogenic conditions. They used di-epoxides and multifunctional epoxies cured with anhydride, amine, or phenol. As part of the conducted research, it was found that linear polymers with a two-dimensional structure show high performance even under cryogenic temperatures. This study showed how important structure control is for optimizing epoxy systems in cryogenic applications. Guo’s team [20] conducted experiments to assess the thermal stability, mechanical properties, and liquid oxygen compatibility of epoxy systems. Cured DDM (4,4′-diaminodiphenyl methane) epoxy resins were found to be most suitable for liquid oxygen. This study offers valuable data for designing epoxy systems for cryogenic tanks. Brem [21] examined the thermal expansion, elastic, plastic, and fracture properties of four epoxy systems used in high-field superconducting magnets at room and cryogenic temperatures. Fracture toughness depends on molecular characteristics beyond van der Waals forces. The findings aid in selecting suitable epoxies for the construction of superconducting magnets and provide guidance for designing cooling/heating systems to mitigate thermal stress damage. In his scientific work, Sindhu [22] studied the effect of low temperature on the resin (DGEBA/TETA) for 90 days, which is commonly used as an adhesive material. It was found that low temperatures change the intra/intermolecular vibration mechanism of the material. Zhou [23] modified the epoxy composition for a liquid oxygen (LOX) fuel tank by adding a phosphor flame retardant and a brominated epoxy resin. This research proved that the composite material is an attractive polymeric material for composite LOX tanks. The Haight research group [24] developed an epoxy resin that is suitable for making lightweight composite materials of a specific diameter for manufacturing tanks. The novel resin was resistant to deformation and microcracking under cryogenic temperatures. 

According to the current state of knowledge [25], analysis of the damage mechanisms of fiber-reinforced polymer composites under cryogenic conditions is limited and the relationship between their cryogenic microscopic behavior and macroscopic properties needs to be more thoroughly investigated. Epoxy resins, due to their relatively low cost, easy availability, high mechanical strength, stiffness, and good chemical resistance, are used in the aerospace industry, space industry, and in the production of gas tanks. At cryogenic temperatures, these resins become quite brittle and prone to cracking. Therefore, the resin needs to be appropriately modified to improve its cryogenic mechanical properties by introducing additional reinforcing agents, such as hardeners, thermoplastics, and inorganic fillers [26]. Thus, the current study is designed to address this shortcoming. 

Although there are many studies in the scientific literature, it has been observed that there is no systematic research [27] investigating the impact of certain parameters on the behavior of composite laminates or directly comparing the behavior of aerospace-grade GFRP and CFRP with the same epoxy matrix in quasi-static tests.

Our preliminary results [28] suggested a notable dependence of the composite response on the type of resin and hardener combinations both at room temperature and at low temperatures. This is manifested in both the offset strain at which degradation started to appear (the initiation of damage) and the rate and amount of degradation at each strain point (the accumulation of damage). Therefore, in the current study, more commercial systems were studied and compared to a newly developed resin believed to have higher resistance to degradation at low temperatures. The new composite material (EP_AD_1) utilizes a resin modified by phosphonium compound catalysts. Thus, we anticipate that it could perform better at low temperatures in terms of damage tolerance, offering a longer service life. 

## 2. Materials and Methods

### 2.1. Materials

The selection of epoxy resins and hardeners was based on their suitability for the product line and manufacturing methods currently employed at IZOERG company (Gliwice, Poland). The following epoxy resins were used as matrices in the developed composite materials:

- EPIDIAN 11M80 (Sarzyna Chemical, Nowa Sarzyna, Poland);
- YD-128 (Aditya Birla Chemicals, Rayong, Thailand);
- YDPN 638A80 (Kukdo, Seoul, Republic of Korea).

The resins were modified with DICY, DDS, AN70/30, and Nowolac P hardeners. The Joint Stock company(Polotsk, Belarus) provided the E-glass fabric with the specifications listed in Table 1, as extracted from the technical datasheet [29]. As part of the conducted research, information about the epoxy resin (EP), the type of epoxy resin (X), and the type of hardener (Y) were used to determine the composite materials. The notation creating the designation looked as follows: EP_X_Y.

In this article, the newly developed materials include EP_AD_1 and EP_2_2.

The technical parameters and epoxy resin designations were extracted from the technical data sheet and are presented in Table 2 [30,31,32].

### 2.2. Composite Manufacturing 

**Figure 1:**
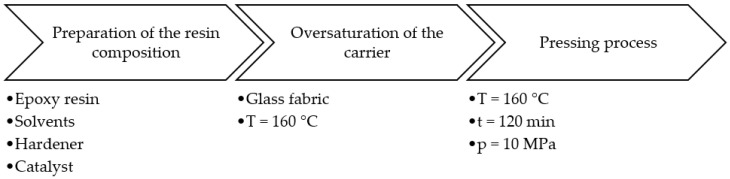
Production scheme of composite material at IZOERG company.

Reference [33] provides comprehensive details on the production of composite sheets. Composite sheets were manufactured at the IZO-ERG S.A. facility in Gliwice, Poland. The process consisted of the following stages outlined in Figure 1:

(1) Resin composition preparation;
(2) Supersaturation of the carrier with the resin composition;
(3) Formation of the board/sheet product using a pressing process.

The selected components of the matrix composition—resin and hardener—were dissolved in the solvent in appropriate proportions. The ingredients were subjected to a mixing process until DICY was completely dissolved. The selection of processing parameters underwent optimization based on internal trials conducted at the IZOERG labs. Thus, further details are subject to confidentiality.

Glass cloth was coated with the EP_X_Y resin compositions utilizing a Hoesch lab coater (Hoesch, VITS U-490, Schwerte, Germany) at a temperature of 160 °C. The carrier was imbued with a suitably selected quantity of matrix, and the carrier–resin system was first cured. The resultant semi-finished product was then divided into sheets measuring 30 cm × 50 cm. These sheets were subjected to compression molding using a Hoesch laboratory press (Hoesch, VITS, Schwerte, Germany) at a temperature of 165 °C for 2 h. Pressing under the aforementioned parameters facilitated the consolidation of the cross-linking effect, culminating in the acquisition of a comprehensive suite of strength properties. The laminate was created through the parallel alignment of 8 sheets of epoxy/glass prepreg in a [0°/90°] orientation (plain weave fabric). Specimens measuring 20 mm × 180 mm were cut from the laminate plate using a guillotine cutter [33]. 

### 2.3. Characterization

**Figure 2:**
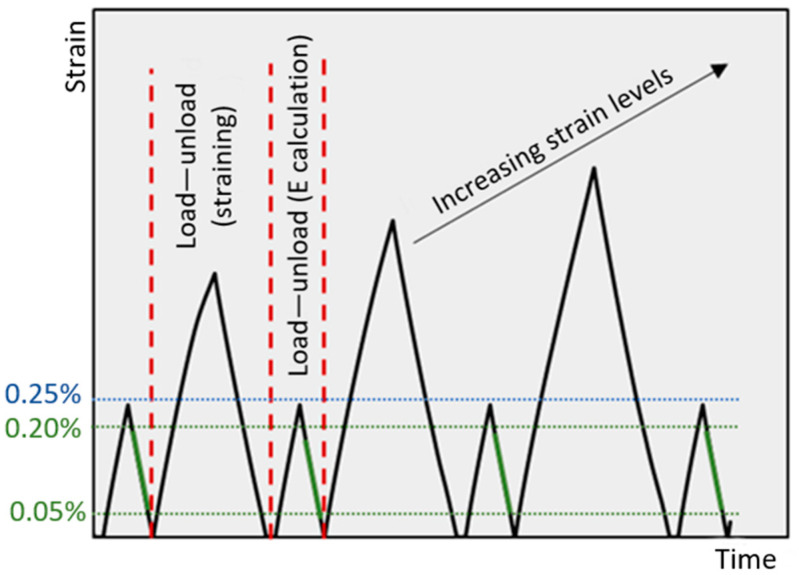
Schematic representation illustrating load application in loading–unloading tests.

**Figure 3:**
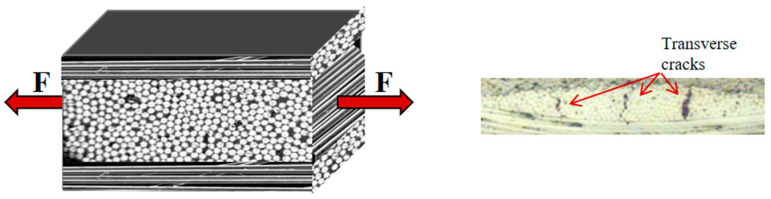
Representation of the cross-ply laminate (**left**) and a micrograph of the laminate with transverse cracks, resulting in stiffness degradation (**right**).

Tensile tests were performed using an Instron 3366 universal testing (825 University Ave, Norwood, MA, USA) machine equipped with a 10 kN load cell and mechanical grips in an environmental chamber (Instron 3119-406 equipped with a Eurotherm 2408 programmer, 825 University Ave, Norwood, MA, USA)). Axial strains were measured using a standard dynamic extensometer (Instron 2620–601, 825 University Ave, Norwood, MA, USA) with a 50 mm base, positioned in the middle of the sample’s gauge length (80 mm). Due to practical limitations, only two specimens for each test were used to obtain the presented results. Quasi-static tests were conducted at room temperature on samples with reduced width (10 mm) to facilitate obtaining failure properties within the limits of load cell capacity. For the low-temperature tests, samples were clamped into the machine inside the chamber, which was connected to a liquid nitrogen tank, and were held at −50 °C for about 30 min before starting the test to ensure thermal saturation. Undesired loads resulting from the contraction of the specimens and the equipment parts during chamber cooling were manually and continuously compensated until thermal equilibrium was reached. The tests were conducted in the displacement control mode at a rate of 2 mm/min. The modulus was determined from stress–strain curves within a linear range from 0.05% to 0.20% strain. In the cyclic loading–unloading tests (L–UL), the samples underwent steps of increasing strain, starting from 0.25% until failure (or up to maximum strain of 1.20%). After each loading step, the samples were unloaded to 15N, the axial strains were zeroed, and a low-level (up to 0.25%) L–UL step was performed from which the modulus was determined. The schematic representation of the loading profile is shown in Figure 2. The tests were conducted in accordance with ASTM D3039 standards [34]. To identify damage (e.g., microcracks), optical microscopy was used. The presence of such damage can result in stiffness degradation in the laminate, which is represented by the ratio of the measured stiffness of the specimen after being subjected to a specific strain level to its initial stiffness (in the undamaged state). Figure 3 shows a representation of a cross-ply laminate on the left and, on the right, a micrograph of a laminate with transverse cracks resulting in stiffness degradation.

## 3. Experimental Results

### 3.1. Tensile Properties

**Figure 4:**
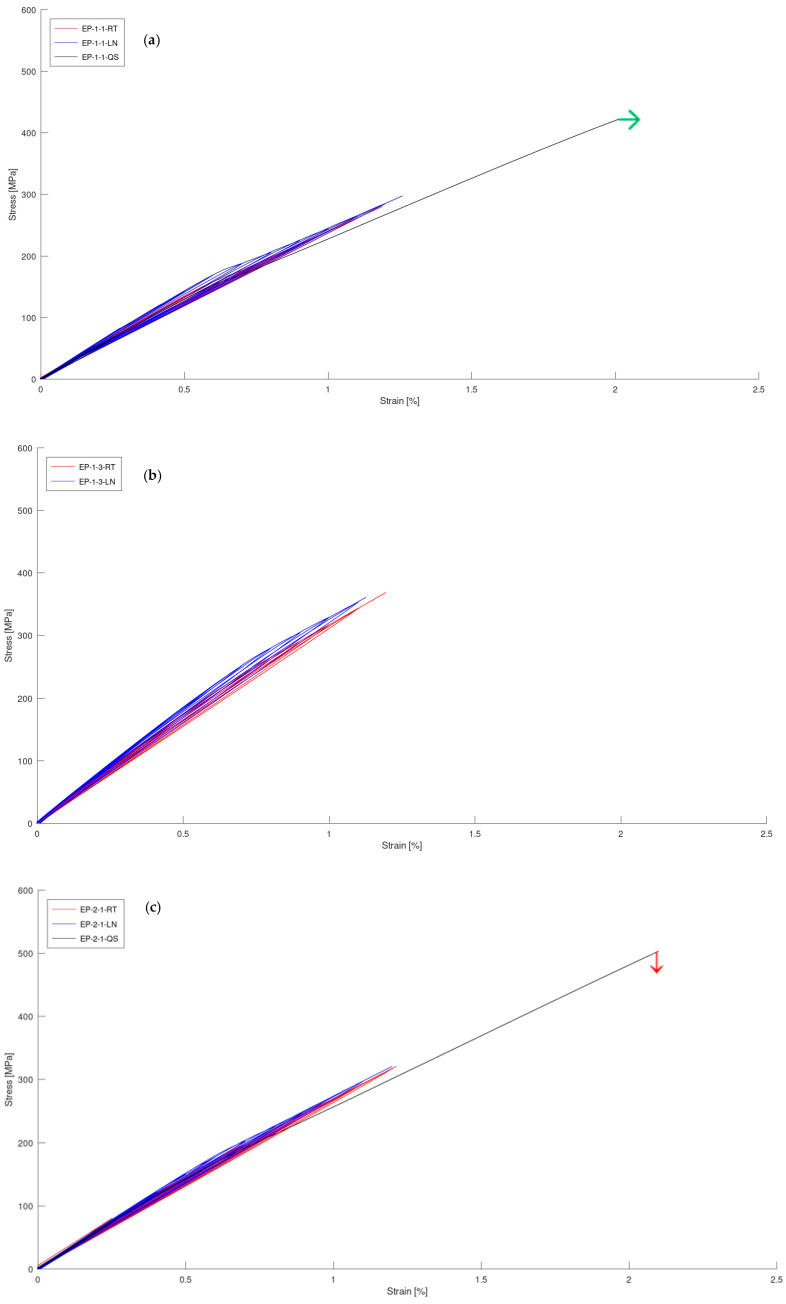

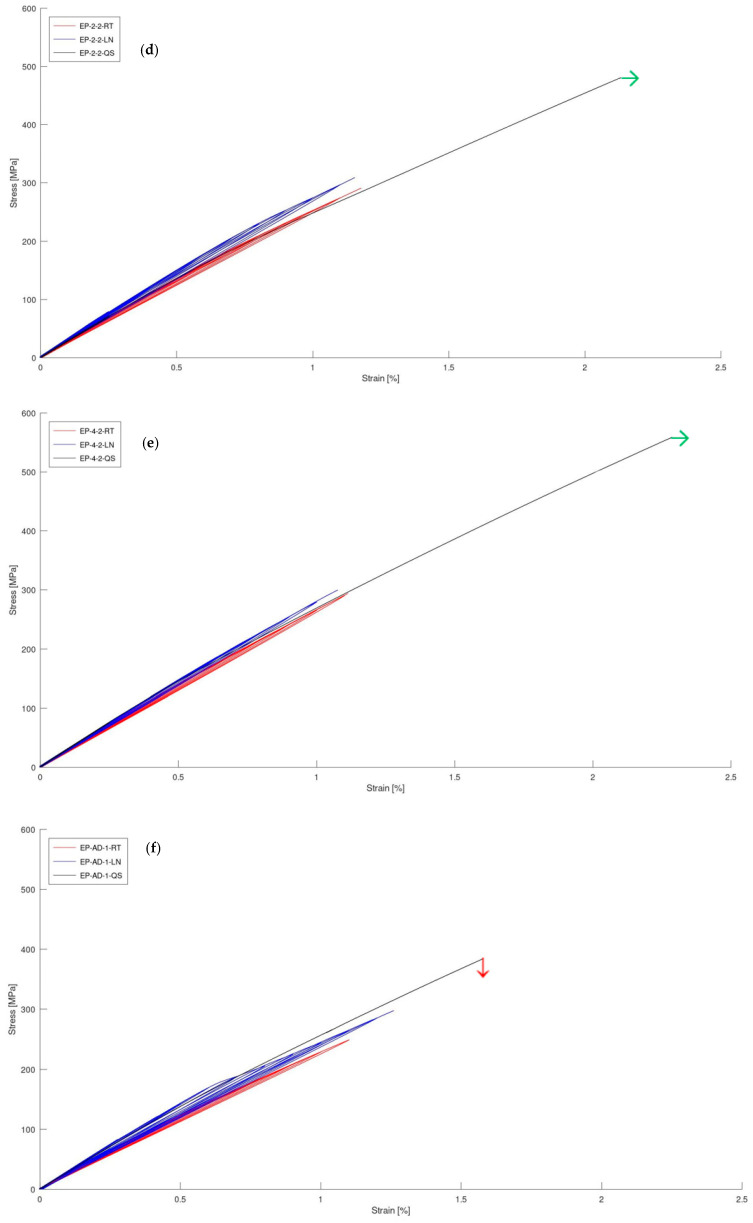
Comparison of loading–unloading stress–strain curves of composite laminates at RT and −50 °C, with curves from quasi-static tensile tests at RT (no data available for EP-1-3 material). The samples that failed during the quasi-static tensile test are marked with a red arrow (↓), whereas for the rest of the materials, the test was interrupted before specimen failure (those curves are marked with a green arrow →). (**a**) EP_1_1, (**b**) EP_1_3, (**c**) EP_2_1, (**d**) EP_2_2, (**e**) EP_4_2, (**f**) EP_AD_1.

**Figure 5:**
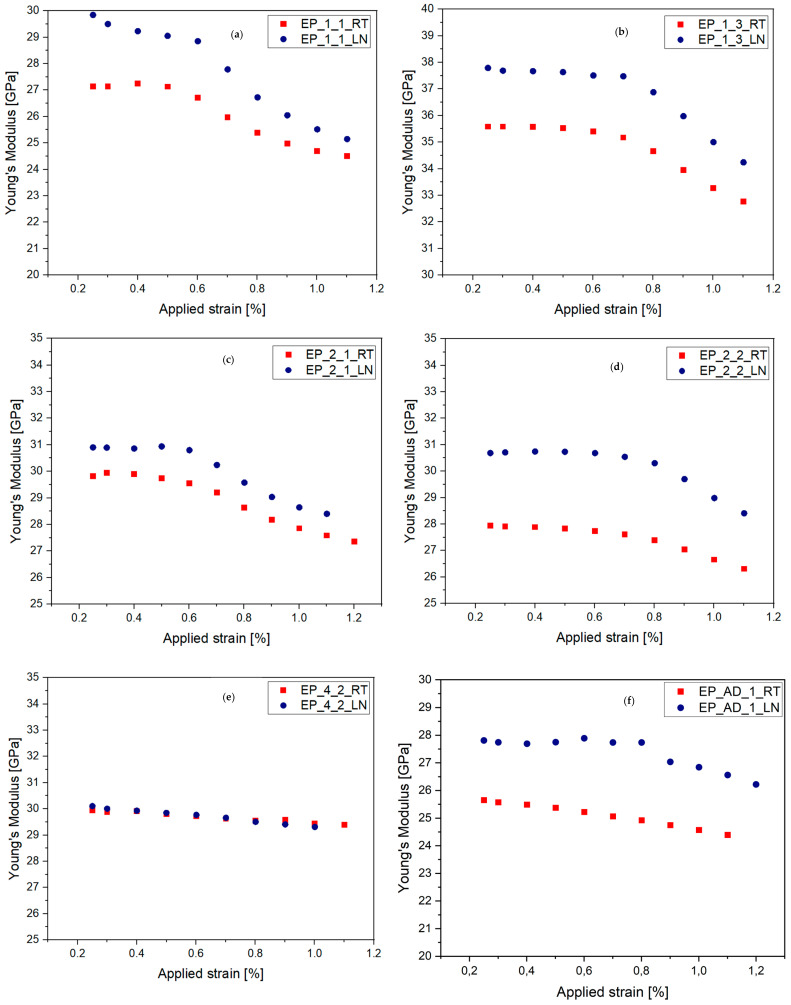
Degradation of Young’s modulus in GFRP laminates with increasing applied strain. (**a**) EP_1_1, (**b**) EP_1_3, (**c**) EP_2_1, (**d**) EP_2_2, (**e**) EP_4_2, (**f**) EP_AD_1.

The aim of the QS tests was to gain a general understanding of the material properties and to extract quasi-static stress–strain curves. These tests provided insights into the maximum stress and initial modulus, which were compared with the stress–strain curve obtained from the gradual L–UL test. Table 3 presents the results of the quasi-static tests conducted on the studied materials. Figure 3 shows the stress–strain curves for all the QS tested materials at RT. Figure 4 presents the stress–strain curves for all the tested materials at room temperature (RT) and at −50 °C. Figure 5 shows the degradation of materials, represented by a reduction in Young’s modulus, as the applied strain increased at RT and −50 °C. For easier comparison, the numerical values of reduced Young’s modulus of laminates at 1.1% for all the tested composites at RT and −50 °C are summarized in Table 3. 

When analyzing the quasi-static results, the highest Young’s modulus value was observed for EP_AD_1 (38.2 GPa), whereas the lowest Young’s modulus was observed for EP_1_1 (27.5 GPa). There was a 32% difference between the highest and lowest values. It is worth noting that both composites shared a common epoxy resin base, but differed in terms of the hardener used. However, EP-1-1 had a greater resin content (according to Table 2), which may be the reason why it had the lowest modulus value. It is important to mention that EP_AD_1 represents a composite with a novel formulation. The inclusion of phosphonium salts in composite materials typically enhances their chemical and thermal stability, making them well-suited for challenging environments. For both EP_2_1 and EP_2_2, bisphenol A was used as the base, but they utilized different hardeners, namely Novolac and DICY. The highest maximum tensile stress was exhibited by EP_4_2, whereas EP_AD_1 exhibited the lowest. This represents a 35.6% difference compared to the weakest composite. It is worth noting that EP_AD_1 failed at 398 MPa, whereas EP_4_2 was loaded to a stress of 540 MPa and still did not fail.

For easier comparison, the numerical values of the strength of the laminates for all the tested composites at RT and −50 °C are summarized in Table 4. 

This is consistent with the literature, which states that the strength of the composite materials improves at low temperatures [9,10,11,12,13]. The highest values, both at room temperature and at low temperatures, were recorded for the EP_1_3, EP_2_1, and EP_4_2 composites. It is important to emphasize that the materials did not fail during the loading–unloading test with a 10 kN load cell; therefore, the strength of the tested materials was estimated to be more than 275 MPa at room temperature and more than 300 MPa at −50 °C. 

**Figure 6:**
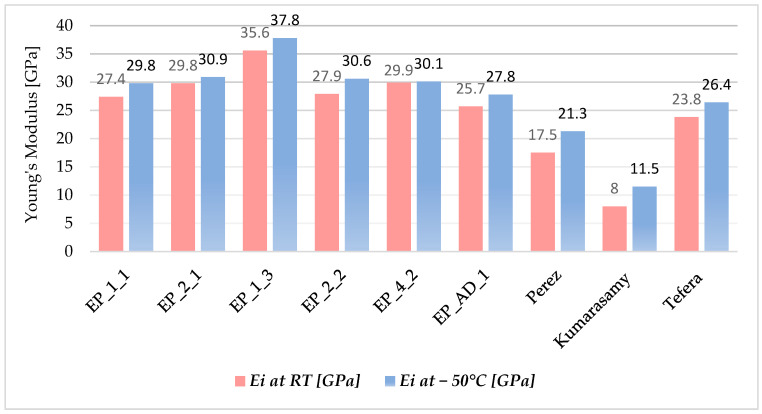
Comparing experimental results (*E*_1.1%_) with data from the literature.

Based on Table 5, it is possible to compare the reduction in stiffness at 1.1% among all the tested composite materials. EP_1_1 exhibited the highest degradation at both room temperature (10.6%) and −50 °C (16.8%), categorizing it as a material with poor crack resistance. EP_4_2 and EP_AD_1 recorded the lowest degradation under both temperature conditions, making them promising options for further research and potential practical use in cryogenic environments. The percentage degradation for EP_4_2 was 2% at room temperature and 2.7% at −50 °C. For the newly developed composite material (EP_AD_1), the degradation was 5.1% and 5.8% at room temperature and −50 °C, respectively. The remaining materials experienced a percentage decrease in Young’s modulus within the range of 5.7–9.3% at both room temperature and low temperatures. To assess the properties of the studied materials with respect to other similar materials, Figure 6 shows a comparison of the composite materials with data from the literature.

In analyzing and comparing the experimental results, it can be concluded that that the studied materials have better properties compared to those found in the literature. It can also be confirmed that low temperatures affect the initiation/accumulation of damage of composite materials and contribute to the phenomenon where Young’s modulus is higher at low temperatures compared to room temperature.

Based on Figure 5, it can be observed that the degradation of stiffness in all the materials exhibited a relatively similar reduction in Young’s modulus, with the exception of EP-4-2. Regardless of the composition and test conditions, a decrease in stiffness was recorded at approximately 1.1% of the applied strain, reaching 3–10% for all the materials. It is known that the decrease in stiffness is directly related to the amount of damage to the composite. Therefore, the damage parameter can be defined as the ratio of the measured stiffness (E) after each maximum strain level to the initial stiffness (*E_i_*) of the undamaged sample (*d = E/E_i_*). If there is no damage, then *d =* 1, but once damage is initiated and microcracks are present, then *d <* 1. This means that at the current stiffness decrease (10%), the damage parameter *d ≈* 0.9. Using data from the scientific literature, it can be inferred that the value of *d =* 0.9 may correspond to a crack density ranging from one to six cracks per 1 cm, depending on the thickness of the transverse layer and the ratio of thickness between the longitudinal and transverse layers [28,35,36]. There are differences in stiffness degradation between the materials/test conditions, but these could not be adequately analyzed because of the limited number of samples tested. Due to these limitations, it was not possible to quantify the damage (crack density) properly at this point. This is because, in the studied material, the cracks in the 90 layers were scattered throughout the whole laminate, and it is technically very challenging to quantify this damage.

There was one noticeable difference between the laminates tested at RT and −50 °C; stiffness began to decrease after 0.6% of applied strain for the laminates tested at RT, whereas, for the materials subjected to −50 °C, Young’s modulus began to change after 0.7%. This indicated that the offset strain for crack initiation may be higher for laminates tested at −50 °C. The polymer also tends to be more brittle at low temperatures. Therefore, it would be expected that cracks in laminates at lower temperatures would appear earlier and more easily. Moreover, the thermal stresses in the 90 layers at low temperatures should also be higher than at RT. This contradiction with the expected/known mechanisms should be investigated further by calculating the thermal stresses in different layers and by performing more experimental work on these materials [28,35,36].

### 3.2. Microscope Observation

**Figure 7:**
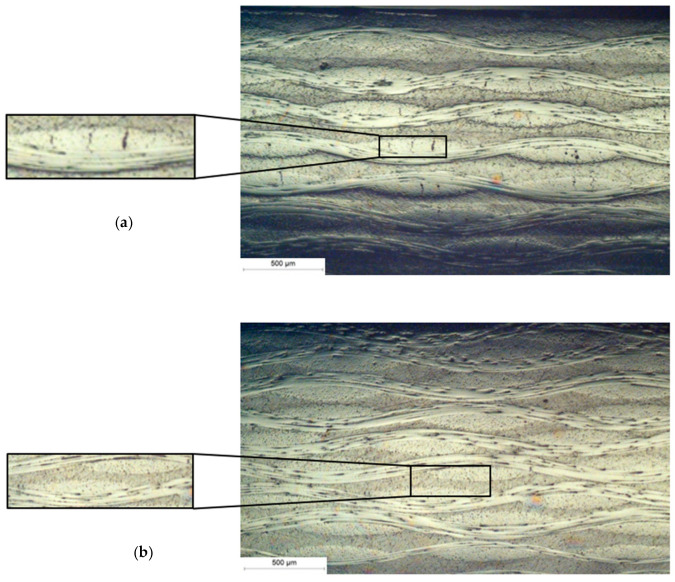
Optical micrographs showing the composite microstructure and microcracks in the transverse bundles. (**a**) the presence of cracks in the selected structure, (**b**) no cracks in the selected structure.

Figure 7 provides insight into the microstructure of laminates with a thickness of 1.5 mm and the presence of transverse microcracks, as seen in the optical micrographs. Although the stiffness of the laminates decreased by approximately 2–16%, as seen in Figure 3, the amount of visible microcracks was not significant, as can be observed in the micrographs. In order to demonstrate examples of microcracks and their destribution accross the material, selected images are presented in Supplementary Materials. This may be because the damage is spread throughout of all the layers with fiber orientations transverse to the loading direction. When the cross-ply laminate contains thick 90 layers, the damage (transverse cracks) is much more visible and it was easier to quantify. No other (more severe) types of damage were detected, such as delamination or fiber fractures.

Under cryogenic conditions, laminates are susceptible to microcracks that lead to destruction of the material’s structure. The cause of these microcracks is the concentration of stress between the laminates and between the fiber and the matrix due to the differing values of the thermal expansion coefficient [37]. Several references confirm that to understand the failure mechanism at sub-zero temperatures, it is necessary to examine the material structure using scanning electron microscopy (SEM)/optical microscopy. Sethi [38] compared the structures of GFRP composite materials at −100, −50, and room temperatures. The most noticeable changes were observed at −100 °C, where numerous crack initiations and laminate delamination were visible.

## 4. Conclusions

In this study, loading–unloading tests were performed to investigate the mechanical properties of GFRP composite laminates at room temperature and −50 °C, as well as the degradation of Young’s modulus under these conditions. The obtained data also include properties of materials obtained from quasi-static tensile tests. Microscopic images were also captured to visualize the locations of damage.

It may be concluded that materials with different epoxy matrices show rather large differences in initial stiffness. Moreover, there are also differences in the degradation of stiffness during loading between different materials.

We demonstrated that the laminate tested at −50 °C showed somewhat higher initial stiffness compared to laminates tested at RT, which aligns with results found in the literature. The effect of temperature is evident in the stiffness degradation at −50 °C, which is more significant and slightly faster compared to RT (except for EP_1_1, where this difference is rather significant). However, the type of damage observed was the same in all cases—only matrix transverse cracks were detected.

In particular, based on actual numbers, the following observations were made:

- The decrease in stiffness in EP_1_1 was approximately ≈10% at RT and ≈17% at −50 °C, which indicated that significant damage was induced in these laminates. The rest of the materials exhibited a lesser degree of stiffness degradation (<10%).
- Composites, such as EP_4_2 and the newly developed material EP_AD_1, exhibited the lowest decrease in Young’s modulus at room temperature and at −50 °C. These materials are being considered for further research at −196 °C and are potentially the best candidates for composites suitable for use under cryogenic conditions.

To conduct a more comprehensive analysis of different matrix formulations with respect to damage initiation and accumulation, there is a need to quantify the number of microcracks under different applied strains. This may be accomplished through direct optical microscopy or by means of analytical modeling. The use of analytical modeling may be more advantageous due to the complexities in experimentally quantifying damage in the studied materials. Existing analytical models allow for relatively accurate predictions of stiffness reduction as a function of crack density. This work is considered a continuation of the presented study.

## Figures and Tables

**Table 1:** Technical parameters of the Joint Stock company 7628 E-type glass fabric.

Parameter	Value
Weight	205 g/m^2^
Weave type	Plain weave
Glass type	“E”
Fiber volume fraction	55–60%

**Table 2:** Characteristics of matrix materials and the designation of composite materials.

Symbol	Epoxy Resin Type	Viscosity [Pa × s]	Epoxy Weight Equivalent (EEW) [g/eq]	Hardener (Y)	Fluidity [%]	Resin Content wt/wt [%]
EP_1_1	YDPN 638 A 80	0.1–0.4	170–190	Novolac	24.6	38
EP_2_2	YD-128	0.011–0.013	182–192	DICY	13	33.6
EP_2_1	YD-128	0.011–0.013	182–192	Novolac	17.9	35.5
EP_1_3	YDPN 638 A 80	0.1–0.4	170–190	DDS	13.1	34.6
EP_4_2	EPIDIAN 11M80	1–5	200–215	DICY	21	34
EP_AD_1	YDPN 638 A 80	0.1–0.4	170–190	AN 7030	28	32.7

**Table 3:** Properties of composite laminates at RT from quasi-static tensile tests.

Symbol	E [GPa]	Maximum Stress [MPa]	Maximum Strain [%]
EP_1_1	27.5	422	2.01
EP_2_1	29.9	503 *	2.09
EP_2_2	27.8	480	2.13
EP_4_2	30.2	539	2.33
EP_AD_1	38.2	398 *	1.58

^*^ Specimens were loaded until failure. The test for the rest of the materials was interrupted because the load reached the maximum capacity of the testing equipment.

**Table 4:** Maximum stress of composite laminates in different conditions.

Material	Maximum Stress at RT [MPa]	Maximum Stress at −50 °C [MPa]
EP_1_1	280.4	297.7
EP_2_1	320.7	320.3
EP_1_3	368.8	361.2
EP_2_2	291.1	308.8
EP_4_2	294.1	299.9
EP_AD_1	275.3	305.6

**Table 5:** Young’s modulus of composite laminates in different conditions at 1.1%.

Material	*E_i_* at RT [GPa]	*E*_1.1%_ at RT [GPa]	Δ*E* at RT [%]	*E_i_* at −50 °C [GPa]	*E*_1.1%_ at −50 °C [GPa]	Δ*E* at −50 °C [%]
EP_1_1	27.4	24.5	10.6	29.8	24.8	16.8
EP_2_1	29.8	27.3	8.4	30.9	28.4	8.1
EP_1_3	35.6	32.8	7.9	37.8	34.3	9.3
EP_2_2	27.9	26.3	5.7	30.6	28.4	7.2
EP_4_2	29.9	29.3	2.0	30.1	29.3	2.7
EP_AD_1	25.7	24.4	5.1	27.8	26.2	5.8

## Data Availability

Data are contained within the article.

## Supplementary Materials

The following supporting information can be downloaded at: https://www.mdpi.com/article/10.3390/ma17010016/s1, Figure S1: Optical micrographs showing the composite microstructure and microcracks in the transverse bundle; Table S1: Optical micrographs showing the composite microstructure and microcracks in the transverse bundle.
