# Supplementary material for: Effect of Thermomechanical Loading at Low Temperatures on Damage Development in Glass Fiber Epoxy Laminates
Source: Materials (Basel). 2023 Dec 20;17(1):16. doi: 10.3390/ma17010016 (PMC10779879; doi:10.3390/ma17010016)
Supplement: Supplementary file 1 [file materials-17-00016-s001.zip › materials-2748896-supplementary.pdf]

materials

Effect of Thermomechanical Loading at Low Temperatures on Damage Development in Glass Fiber Epoxy Laminates

A. Krzak*[1], Z. Al-Maqdasi[2], A.J.Nowak[1], R. Joffe[2]

[1] Scientific and Didactic Laboratory of Nanotechnology and Material Technologies, Silesian University of Technology, Gliwice, Poland
[2] Department of Engineering Sciences and Mathematics, Luleå University of Technology, Luleå, Sweden
* Correspondence: anna.krzak@polsl.pl

Supplementary Materials

In order to demonstrate examples of microcracks and their distribution across the material, selected images have been included in the supplementary document for reference.

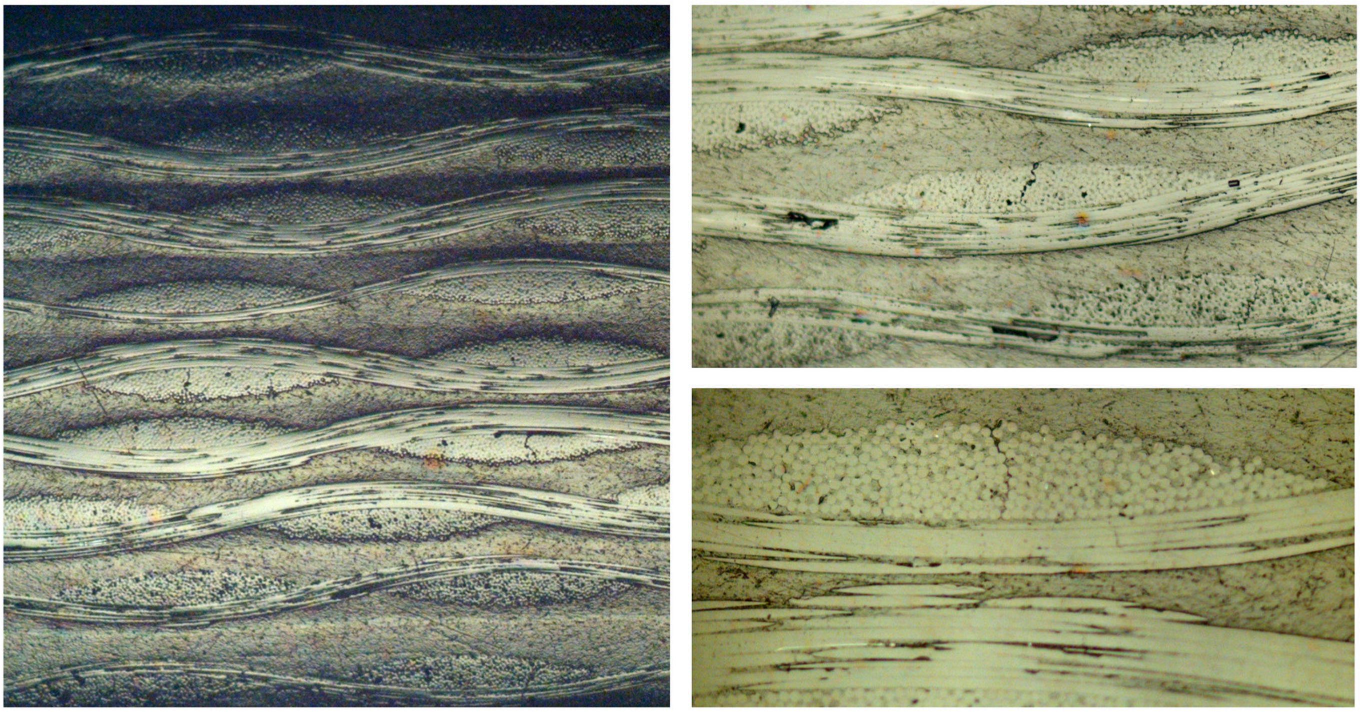

Figure S1 *Optical micrographs showing the composite microstructure and microcracks in the transverse bundle.*

Table S1 *Optical micrographs showing the composite microstructure and microcracks in the transverse bundle*

	**RT**	**- 50°C**
**EP_1_1**	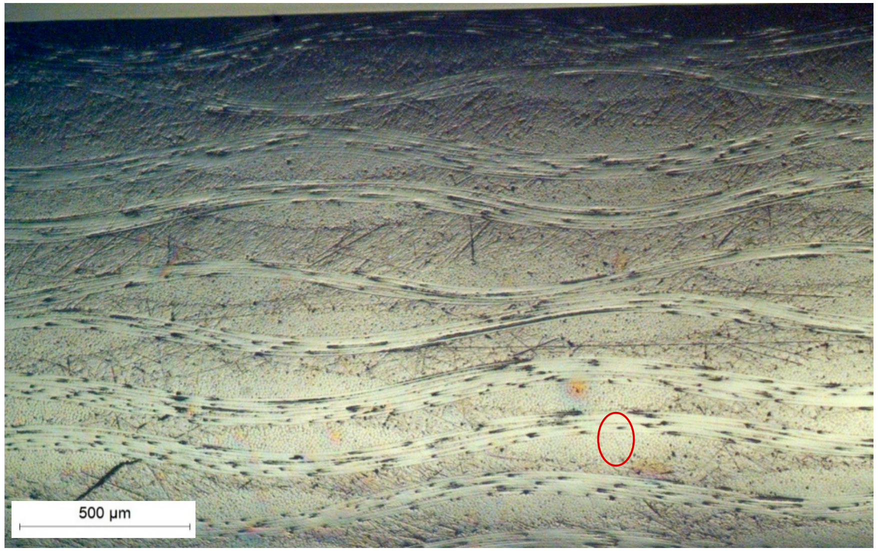	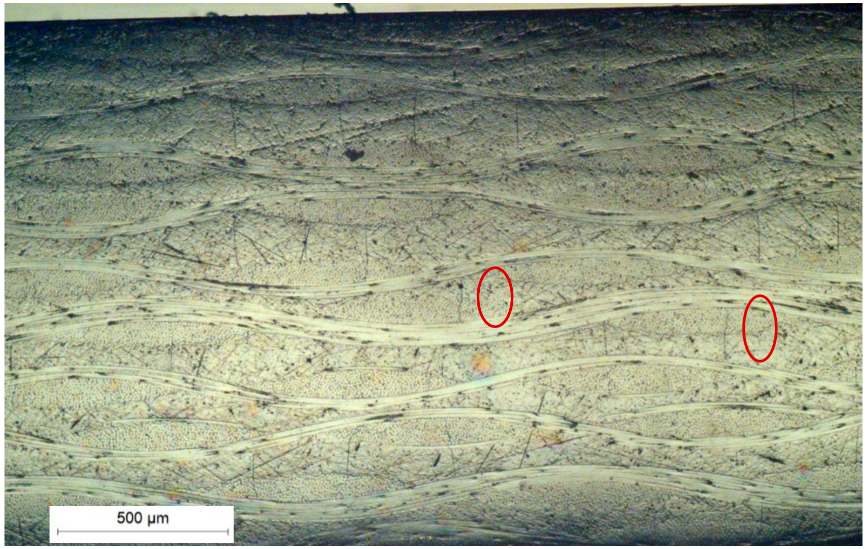

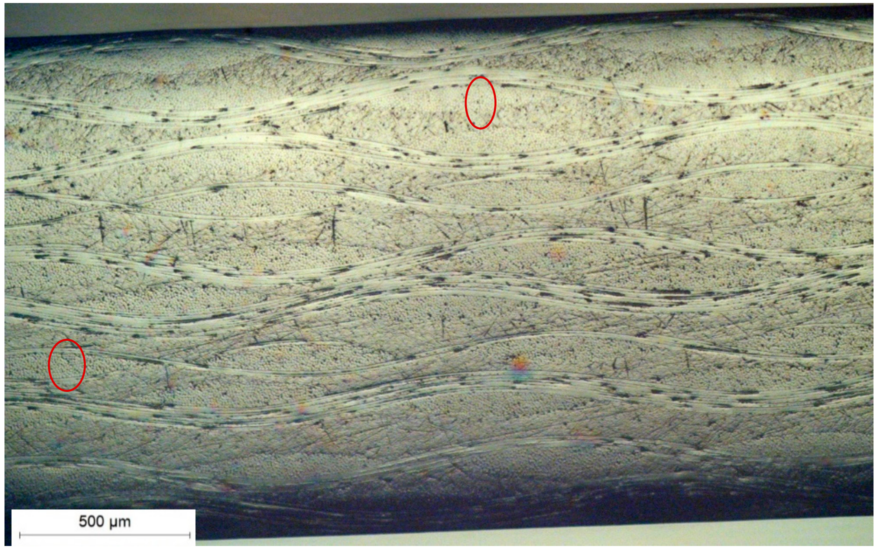

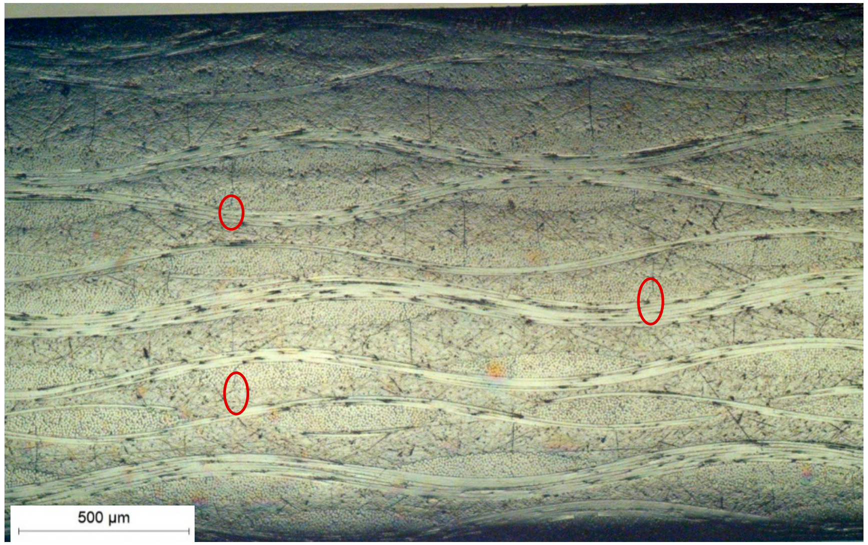

EP_1_3

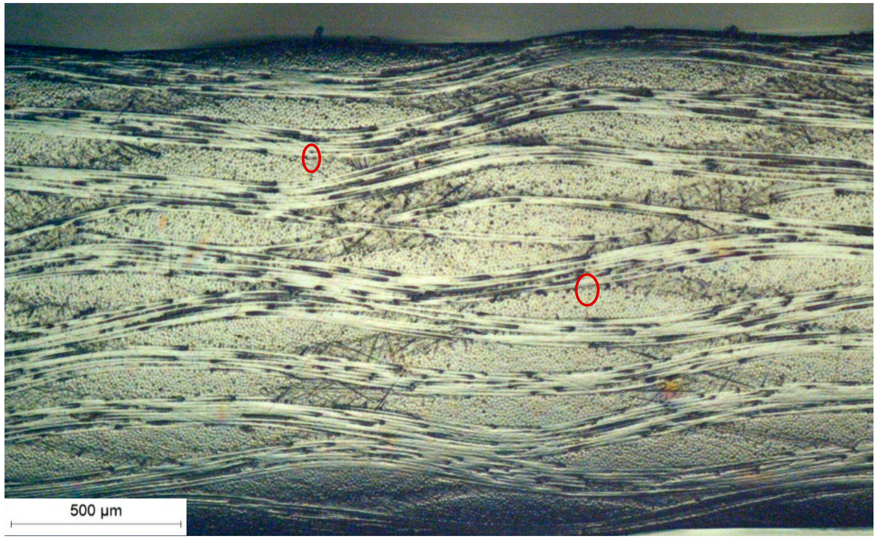

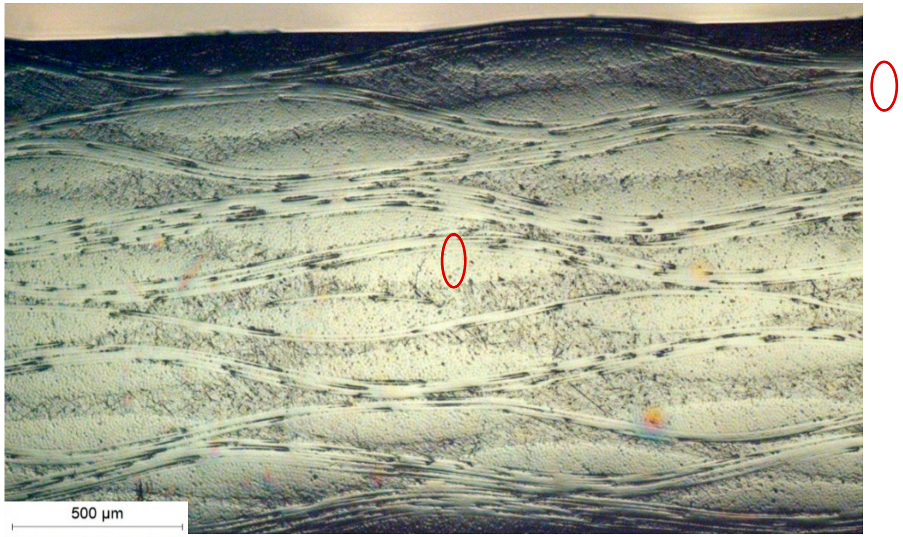

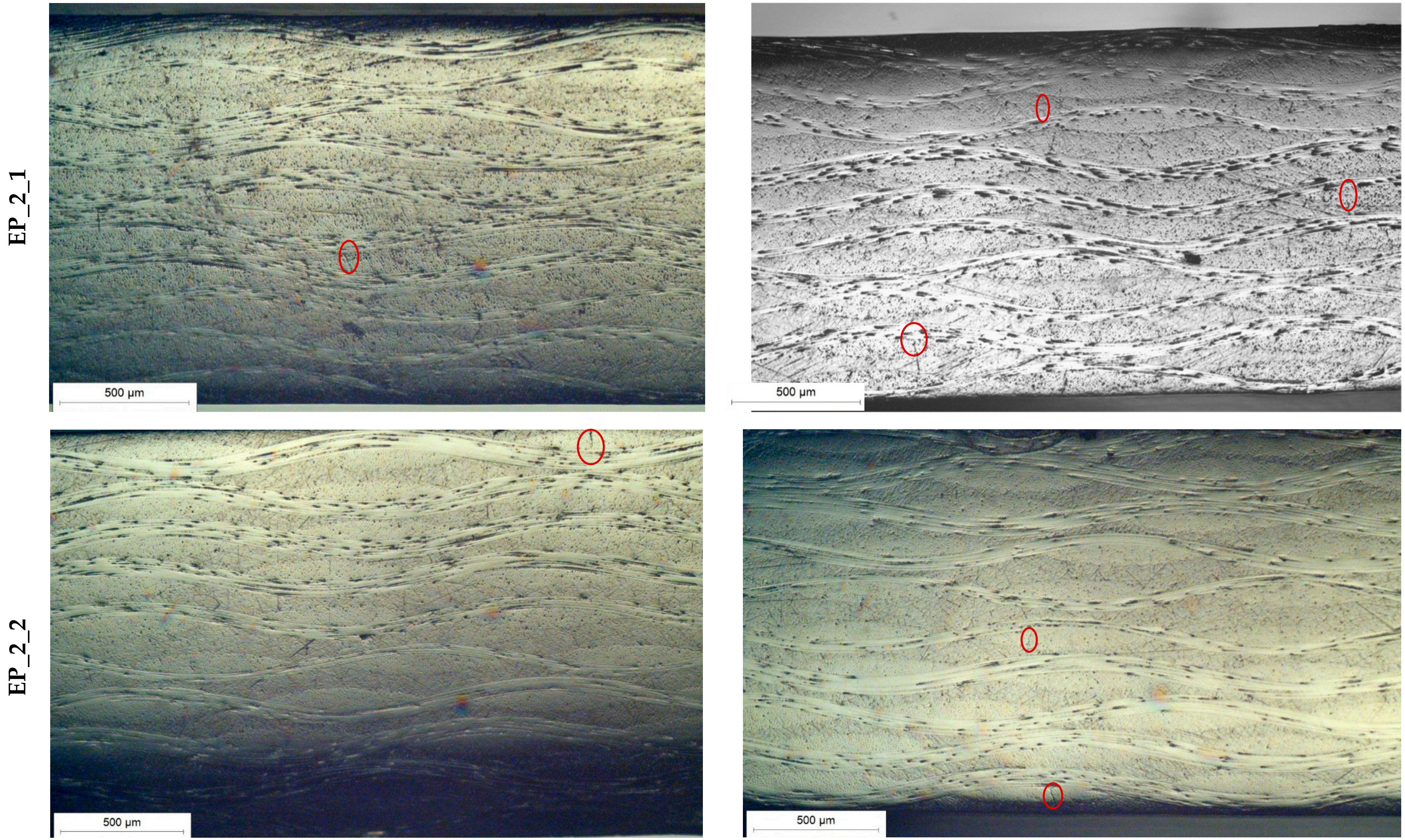
EP_2_1
EP_2_2
500 µm
500 µm
500 µm
500 µm

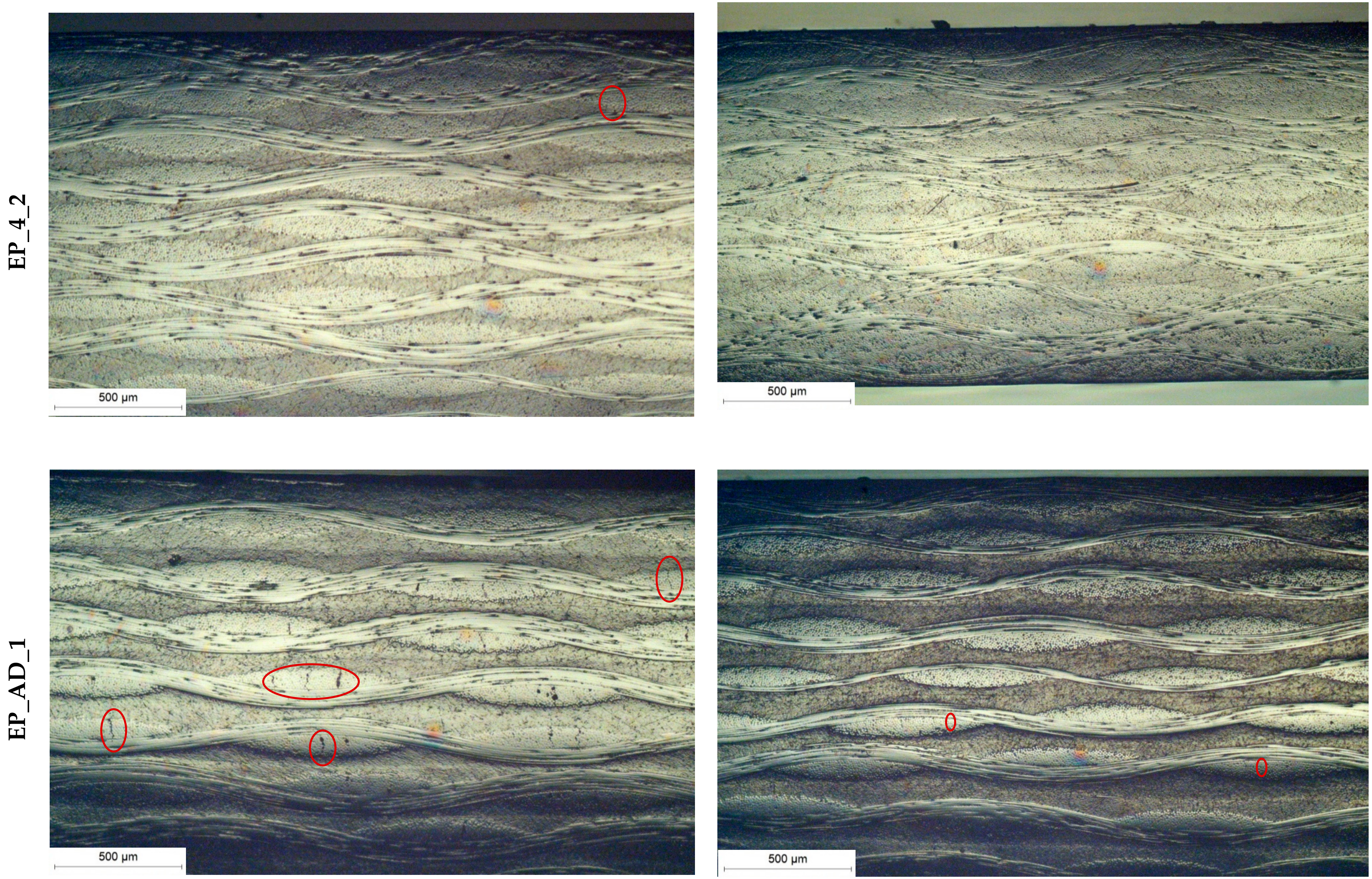
EP_4_2
500 µm
500 µm
EP_AD_1
500 µm
500 µm
